# Gabapentinoids Associated With Lower Explantation Rate in 203 Patients With Spinal Cord Stimulation for Failed Back Surgery Syndrome

**DOI:** 10.1093/neuros/nyab242

**Published:** 2021-07-16

**Authors:** Mette Nissen, Tiina-Mari Ikäheimo, Jukka Huttunen, Ville Leinonen, Henna-Kaisa Jyrkkänen, Mikael von und zu Fraunberg

**Affiliations:** Neurosurgery of KUH NeuroCenter, Kuopio University Hospital, Kuopio, Finland; Faculty of Health Sciences, School of Medicine Institute of Clinical Medicine – Neurosurgery, University of Eastern Finland, Kuopio, Finland; Neurosurgery of KUH NeuroCenter, Kuopio University Hospital, Kuopio, Finland; Neurosurgery of KUH NeuroCenter, Kuopio University Hospital, Kuopio, Finland; Neurosurgery of KUH NeuroCenter, Kuopio University Hospital, Kuopio, Finland; Faculty of Health Sciences, School of Medicine Institute of Clinical Medicine – Neurosurgery, University of Eastern Finland, Kuopio, Finland; Neurosurgery of KUH NeuroCenter, Kuopio University Hospital, Kuopio, Finland; Neurosurgery of KUH NeuroCenter, Kuopio University Hospital, Kuopio, Finland; Faculty of Health Sciences, School of Medicine Institute of Clinical Medicine – Neurosurgery, University of Eastern Finland, Kuopio, Finland

**Keywords:** Spinal cord stimulation, Neuropathic pain medication, Failed back surgery syndrome

## Abstract

**BACKGROUND:**

Spinal cord stimulation (SCS) is an effective treatment in failed back surgery syndrome (FBSS). The effect of neuropathic pain medication use on SCS outcome is poorly understood.

**OBJECTIVE:**

To study the effect of gabapentinoid use on SCS outcome measured by trial success, explantation rate and opioid dose reduction during a 2-yr follow-up.

**METHODS:**

The study cohort included 203 consecutive FBSS patients who underwent SCS in a single tertiary center during January 1997 to March 2014. Purchase data of gabapentinoids, opioids, tricyclic antidepressants, serotonin and noradrenaline reuptake inhibitors, and benzodiazepines during January 1995 to March 2016 were retrieved from national registries.

**RESULTS:**

In multivariate Cox regression analysis, patients using gabapentinoids had significantly fewer explantations during the 2-yr follow-up (hazard ratio [HR] 0.2, 95% CI 0.04-0.81, *P =* .03). In contrast, patients with opioid use of >40 morphine milligram equivalent before implantation had significantly more explantations (HR 6.7, 95% CI 2.5-18, *P <* .01). In bivariate logistic regression analysis adjusted for patient specific factors, year of SCS implantation, use of neuropathic pain medication, opioids, and benzodiazepines, patients using gabapentinoids significantly more often discontinued opioids or reduced their dose by more than 50% during the 2-yr follow-up (odds ratio 5.7, 95% CI 1.4-23, *P = .*015).

**CONCLUSION:**

The use of gabapentinoids was associated with a significantly lower spinal cord stimulator explantation rate and a higher chance of opioid discontinuation or >50% dose reduction. This indicates that patients with SCS could benefit from concomitant use of gabapentinoids. Prospective randomized trials are warranted to verify this hypothesis.

**ABBREVIATIONS**
CDC: Centers for Disease Control and Prevention
DDD: defined daily dose
FBSS: failed back surgery syndrome
GABA: gamma-aminobutyric acid
IPG: implantable pulse generator
KUH: Kuopio University Hospital
MME: milligrams of morphine equivalent
SCS: spinal cord stimulation
SNRI: serotonin and noradrenaline reuptake inhibitor
TCA: tricyclic antidepressant

Failed back surgery syndrome (FBSS) is a term used to describe patients with persistent pain after lumbar spinal surgery.^1^ The typical symptom is pain in the lower extremities and/or chronic back pain, leading to low health-related quality of life.^2^ Failure rates are 30% to 46% for lumbar fusion and 19% to 25% for discectomy.^3,4^ FBSS lacks curative treatment, and its complexity suggests a multidisciplinary team approach to optimize the outcome.

The pharmacological treatment of FBSS with a predominant neuropathic radicular component is based on gabapentinoids and antidepressants.^5^ Gabapentinoids prevent trafficking of the calcium channel complex to the plasma membrane via their binding to the α2δ subunit, which is functionally upregulated following nerve injury in animal models and may contribute to the development of hyperalgesia and allodynia.^6^ This indicates that voltage-gated calcium channels are a key pain target.^7^

 In spinal cord stimulation (SCS), epidural electric stimulation to dorsal columns in the spinal cord elicits a sensation of paresthesia in the corresponding dermatomes.^8^ It is a safe and cost-effective treatment for selected patients not responding to conventional pharmacological treatment for FBSS.^9-11^

We present a retrospective analysis of gabapentinoid use among 203 consecutive FBSS patients treated with SCS in a single tertiary hospital during a 17-yr period. We studied the effect of gabapentinoid use on SCS outcomes measured by trial success, explantation rate, and opioid dose reduction in patients with continuous SCS use during a 2-yr follow-up.

## METHODS

### Study Population

**FIGURE 1.**
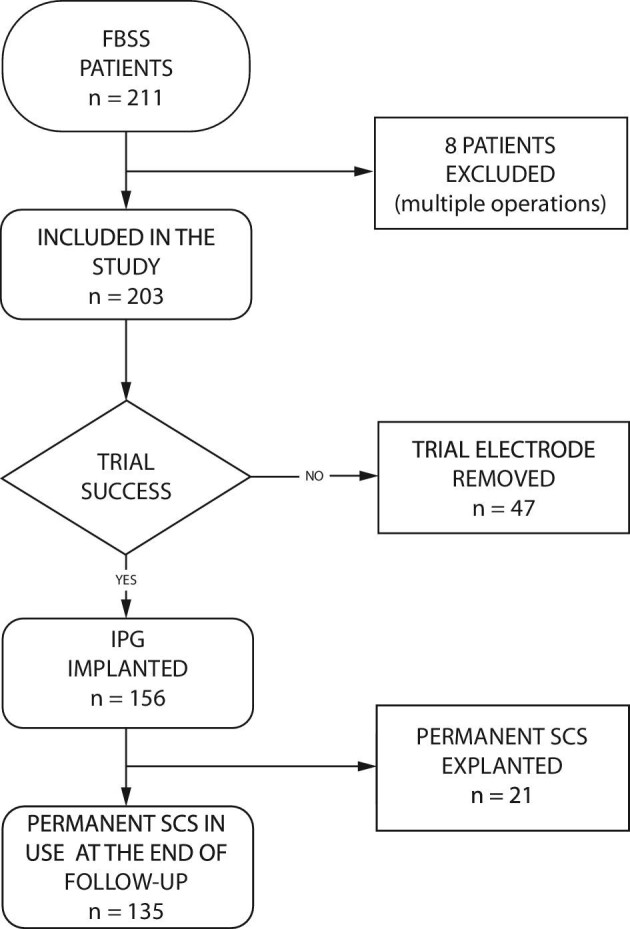
Flow chart. Flow chart of 211 consecutive FBSS patients who underwent SCS with a surgically implanted paddle lead from 1997 to 2014 at Kuopio University Hospital.

Kuopio University Hospital (KUH) is a tertiary center solely providing full-time acute and elective neurosurgical services for the 850 000 person catchment population in Finland. The study group consists of all consecutive 211 patients (Figure 1) who underwent an SCS trial with surgical paddle lead at KUH between January 1, 1997, and March 31, 2014. The stimulation paradigm was solely tonic stimulation.

FBSS was defined as radicular leg pain with or without lumbar pain after one or several lumbar surgeries due to spinal stenosis or disc herniation. The diagnosis was made by a pain specialist, orthopedic surgeon, or neurosurgeon. Conservative treatment according to best practice was initiated, including oral analgesics and physical therapy.

### SCS Implantation

The SCS paddle-lead electrode (Resume 3586, Symmix 3982, Specify 2×4 3998, or Specify 5-6-5 39 565, Medtronic, Dublin, Ireland) was microsurgically implanted into the epidural space under direct visual control with the operating microscope under general anesthesia. The implantation techniques have previously been described.^12^

Implantable pulse generator (IPG, model 7425, model 37 703, model 7427V, model 37 702, or model 97 702, Medtronic, Dublin, Ireland) was implanted after trial (mean duration 7 d, range 0-21) in patients, who reported adequate pain relief and paresthesia, covering of most of the leg pain area. Global patient satisfaction instead of a formal percentage of pain relief was used as a criterion for permanent IPG implantation. All patients receiving an IPG had an outpatient clinic visit 2 to 4 mo (mean 105 d) postsurgery and when needed (total 378 visits).

Overall, 211 patients underwent SCS trial during the study period. Permanent SCS was implanted in 164 patients, and 47 patients had only trial phase. After SCS implantation, 8 patients had their SCS device explanted and reimplanted for infection (n = 4) or electrode type change (n = 4), and they were excluded from subsequent analyses (Figure 1). The SCS device was permanently explanted in 21 (13%) patients, while 135 patients continued to use SCS throughout the 2 yr of follow-up.

### Patient Demographics

Detailed and structured data collection was performed from the medical records. Questionnaires regarding quality of life, pain intensity, or physical performance status were not widely available. Untreated depression and other serious psychiatric illnesses were considered a contraindication for SCS. Informed consent was not required by Finnish legislation, because the study was based on registry data, and patients were not contacted.

### Medication Data and Classes of Medicine

We retrospectively retrieved medication purchase data patients from the Social Insurance Institution of Finland, using the Anatomical Therapeutic Chemical Classification System of the World Health Organization (WHO). The Finnish Social Insurance Institution upholds a prospective registry consisting of prescribed medications, prescription dates, medication purchase dates, amounts, and prices of all prescription medications of citizens living in Finland. We studied the purchasing data for gabapentinoids, tricyclic antidepressants (TCA), opioids, serotonin and noradrenaline reuptake inhibitors (SNRI), and benzodiazepines. Nonsteroidal anti-inflammatory drugs (NSAIDs) were excluded from this study because approximately half of ibuprofein use, which is the most common NSAID in our country, consists of over-the-counter purchases.

We calculated the total defined daily doses (DDD) for each medicine by multiplying the strength of each tablet by the total purchased package size and dividing the result by the DDD of the medication as defined by the WHO.

Opioids included morphine, hydromorphone, oxycodone, codeine, fentanyl, dextropropoxyphene, buprenorphine, tramadol, and methadone. Their conversion ratios to milligrams of morphine equivalents (MMEs) were derived from the Centers for Disease Control and Prevention (CDC) as follows: 0.15 (codeine), 0.10 (tramadol), 75 (buprenorphine TD), 0.2 (dextropropoxyphene), 1.0 (morphine), 3.0 (methadone), 1.5 (oxycodone), 4.0 (hydromorphone), and 100 (fentanyl TD). The conversion factors for transdermal fentanyl and methadone are used by the Centers for Medicare & Medicaid Services with assumption that one patch delivers the dispensed micrograms per hour over a 24-h day, and 1 mg of parenteral fentanyl is equivalent to 100 mg of oral morphine.^13-17^ For subsequent analyses, opioid users were divided into 2 groups: low-dose group with mean opioid use of <40 MME/day and high-dose group with mean opioid use of >40 MME/day.^18^

Benzodiazepines included diazepam, chlordiazepoxide, oxazepam, potassium clorazepate, lorazepam, clobazam, and alprazolam. Gabapentinoids included gabapentin and pregabalin. TCAs included amitriptyline and nortriptyline. SNRIs included venlafaxine and duloxetine.

We studied the purchase data encompassing the 2 yr before and after SCS device implantation to have a complete history for all patients. Use of medication is defined as 2 or more purchases during the given period.

### Survival Analysis

A Cox regression survival analysis model was used to determine factors associated with explant for ineffective therapy. Results were presented as an adjusted multivariable model with the independent variables of gender, age, number of previous operations, duration of pain, location of pain, instrumented lumbar fusion (yes/no), year of SCS operation (<2004, 2005-2009, >2010), use of benzodiazepines, use of opioids >40 MME/day, use of TCAs, use of SNRIs, and use of gabapentinoids.

### Statistical Analysis

Categorical data have been presented as frequencies and proportions and assessed with chi-square or Fisher's exact tests as appropriate. Continuous data that were not normally distributed were assessed with the Mann-Whitney *U* test. Binary logistic regression analysis was used to compare trial success and opioid dose reduction with gender, age, number of previous operations, duration of pain, location of pain, status of instrumented lumbar fusion, year of SCS operation, use of benzodiazepines, use of opioids >40 MME/day, use of TCAs, use of SNRIs, and use of gabapentinoids as covariates. A linear mixed effect model was used to determine interactions with the following covariates: time (categorical), group, and time * group, with neuropathic pain medication use as the dependent variable. All 2-sided *P*-values < .05 were considered statistically significant.

### Ethical Issues

Study protocol was approved by the Institutional Review Board of KUH.

## RESULTS

### Neuropathic Pain Medication Use During the Follow-up

Of the 203 patients included in the study, 76 (37%) were using gabapentinoids preoperatively, and of these, 26 (34%) were also using opioids over 40 MME/day. Of the 127 patients not using gabapentinoids preoperatively, 25 (20%) patients were using opioids over 40 MME/day. Gabapentinoids were combined with TCA in 20 (26%) patients, and SNRI in 18 (24%) patients. TCA alone was used in 21 (16%) patients, and SNRI alone in 14 (11%) patients. Following a 1-wk trial, the SCS electrode was removed in 47 patients (SCS trial only). Gabapentinoid use was not associated with trial success (Table 1). An internal pulse generator was implanted in 156 patients. Of these, 135 (87%) continued to use SCS throughout the 2-yr follow-up (permanent SCS), and 21 (13%) patients had their device explanted during the follow-up (explanted SCS) (Figure 1). Time trends in prescribed neuropathic medication purchases are shown in Table 2.

**TABLE 1.** Patient Characteristics and Multivariate Analysis of Variables Associated With Spinal Cord Stimulation Trial Success, Explantation Rate, and Opioid Decrease >50%

	SCS trial OR for unsuccessful trial				Explantation (within 2 yr) HR for explant				Opioid discontinued or dose decrease > 50% OR for success			
	IPG implant n = 156	Trial only n = 47	OR (95% CI)	*P*	Permanent n = 135	Explanted n = 21	HR (95% CI)	*P*	Unsuccess n = 52	Success n = 37	OR (95% CI)	*P*
Age	48.5 ± 11	47.4 ± 11	0.99 (0.96-1.0)	.51	48.8 ± 10	46.6 ± 15	0.98 (0.93-1.0)	.33	47.7 ± 10	50.9 ± 9.0	1.0 (0.98-1.1)	.22
Gender												
Female	79 (79%)	21 (21%)	1		67 (85%)	12 (15%)	1		22 (49%)	23 (51%)	1	
Male	77 (75%)	26 (25%)	1.3 (0.64-2.5)	.49	68 (88%)	9 (12%)	0.80 (0.30-2.1)	.65	30 (68%)	14 (32%)	0.33 (0.11-1.1)	.06
Pain location												
Leg	67 (77%)	20 (23%)	1		58 (87%)	9 (13%)	1		24 (69%)	11 (31%)	1	
Leg and back	89 (77%)	27 (23%)	1.1 (0.54-2.3)	.78	77 (87%)	12 (13%)	0.53 (0.18-1.5)	.23	28 (52%)	16 (48%)	2.8 (0.84-9.1)	.10
No. of previous operations	2.4 ± 1.6	2.3 ± 1.3	0.94 (0.73-1.2)	.64	2.3 ± 1.5	2.5 ± 1.9	0.91 (0.66-1.2)	.91	2.3 ± 1.2	2.5 ± 1.5	1.1 (0.70-1.8)	.64
Duration of pain	7.1 ± 6.1	8.6 ± 7.7	1.0 (0.99-1.1)	.12	7.0 ± 6.0	8.1 ± 6.8	1.01 (0.93-1.1)	.87	6.9 ± 10	8.1 ± 6.4	1.0 (0.92-1.1)	.94
Instrumented fusion												
No	102 (76%)	33 (24%)	1		90 (88%)	12 (12%)	1		32 (58%)	23 (42%)	1	
In place or removed	54 (79%)	14 (21%)	0.73 (0.33-1.6)	.43	45 (83%)	9 (17%)	1.6 (0.57-4.6)	.36	20 (59%)	14 (41%)	1.6 (0.42-5.8)	.51
Year of implantation				.70				.04				.28
1997-2004	42 (74%)	15 (26%)	1		37 (88%)	5 (12%)	1		10 (50%)	10 (50%)	1	
2005-2009	50 (76%)	16 (24%)	0.97 (0.40-2.4)	.95	46 (92%)	4 (8%)	0.68 (0.17-2.7)	.58	22 (69%)	10 (31%)	0.37 (0.09-1.6)	.18
2010-2014	64 (80%)	16 (20%)	0.72 (0.30-1.7)	.46	52 (81%)	12 (19%)	3.0 (0.98-9.2)	.054	20 (54%)	17 (46%)	0.33 (0.07-1.4)	.14
Benzodiazepine	*	*			**	**			**	**		
Not in use	130 (77%)	39 (23%)	0.93 (0.38-2.3)		114 (88%)	16 (12%)	1		42 (58%)	31 (43%)	1	
In use	26 (77%)	8 (24%)	0.84 (0.34-2.1)	.88	21 (81%)	5 (19%)	0.89 (0.29-2.7)	.84	10 (63%)	6 (37%)	0.41 (0.07-2.3)	.32
Opioid use over 40 morphine milligram equivalents	*	*			**	**			*	*		
Not in use	119 (78%)	33 (22%)	1		106 (93%)	8 (7%)	1		31 (50%)	31 (50%)	1	
In use	37 (73%)	14 (27%)	1.5 (0.68-3.3)	.31	29 (69%)	13 (31%)	6.7 (2.5-18)	<.01	21 (78%)	6 (22%)	0.20 (0.05-0.87)	.032
TCA					**	**			**	**		
Not in use	124 (77%)	38 (23%)	1		114 (86%)	18 (14%)	1		37 (52%)	34 (48%)	1	
In use	32 (78%)	9 (22%)	0.84 (0.34-2.1)	.84	21 (88%)	3 (12%)	1.8 (0.31-5.4)	.75	15 (83%)	3 (17%)	0.22 (0.04-1.3)	.09
Gabapentinoid					**	**			**	**		
Not in use	94 (74%)	33 (26%)	1		94 (84%)	18 (16%)	1		40 (67%)	20 (33%)	1	
In use	62 (82%)	14 (18%)	0.59 (0.27-1.3)	.18	41 (93%)	3 (7%)	0.18 (0.04-0.81)	.026	12 (41%)	17 (59%)	5.7 (1.4-23)	.015
SNRI					**	**			**	**		
Not in use	133 (78%)	38 (22%)	1		117 (87%)	17 (13%)	1		48 (63%)	28 (37%)	1	
In use	23 (72%)	9 (28%)	1.5 (0.68-3.3)	.41	18 (82%)	4 (18%)	4.2 (0.99-18)	.052	4 (31%)	9 (69%)	3.2 (0.45-22)	.25

SCS, spinal cord stimulation; SNRI, serotonin and noradrenaline reuptake inhibitors; TCA, tricyclic antidepressant.

Patient characteristics and multivariate analysis of variables associated with spinal cord stimulation (SCS) trial success (n = 203, all patients), explantation rate (n = 156, patients receiving permanent SCS device), and opioid decrease >20% (n = 89, patients with opioid use before implantation and SCS in place throughout the 2-yr follow-up). Medication use was defined as 2 or more purchases in a years during the *last year before implantation or ** after implantation.

**TABLE 2.** Total Number of Prescribed Neuropathic Pain Medication Purchases in 203 Patients With SCS Trialed or Implanted During 1997 to 2014

	Pregabalin	Gabapentin	Nortriptyline	Amitriptyline	Venlafaxin	Duloxetine
1997 to 2004	0	22	0	262	0	0
2005 to 2009	263	39	13	432	768	0
2010 to 2014	815	124	233	733	512	154

**FIGURE 2.**
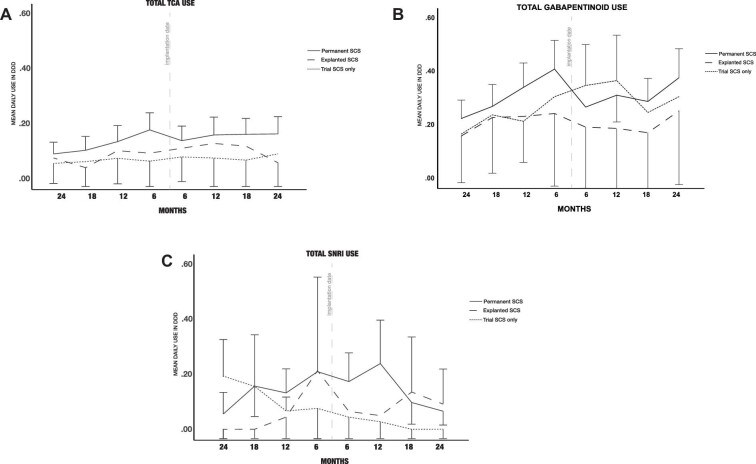
Gabapentinoid use in mean daily defined doses. Mean daily defined dose (DDD) of **A**, tricyclic antidepressants (TCA), **B**, gabapentinoids, and **C**, serotonin and noradrenaline reuptake inhibitors (SNRI) in 24 mo before and after implantation of spinal cord stimulation (SCS) in 203 failed back surgery syndrome (FBSS) patients at Kuopio University Hospital. Trial SCS only = SCS trial only with no permanent implantation, n = 47; permanent SCS = SCS implanted and in use throughout the 2-yr follow-up, n = 135; explanted SCS = SCS implanted, but later explanted during the 2-yr follow-up, n = 21. The mean DDD was calculated as the average of total purchased drugs during the specific 6-mo period (months: 0-6; 6-12; 12-18; 18-24 before and after SCS).

The mean use of gabapentinoids in the 6-mo period before implantation was 0.5 ± 0.7 (mean ± SD) DDD/day in the permanent SCS group, 0.3 ± 0.7 DDD/day in the SCS trial only group, and 0.3 ± 0.6 DDD/day in the explanted group (Figure 2). No significant differences were shown between groups. During the last 6 mo of the follow-up period, the mean use of gabapentinoids was 0.4 ± 0.8 DDD/day in the permanent SCS group, 0.3 ± 0.6 DDD/day in the SCS trial-only group, and 0.3 ± 0.6 DDD/day in the explanted group. No significant differences were shown between groups (linear mixed effect model with time, group, and time * group as fixed variables).

### Gabapentinoid Use and SCS Explantation

Of the 156 patients with SCS devices implanted after trial, 44 (28%) were using gabapentinoids during the 2-yr follow-up, and of these, 11 (25%) were also using opioids over 40 MME/day. Of the 112 patients not using gabapentinoids during follow-up, 31 (28%) patients were using opioids over 40 MME/day. Gabapentinoids were combined with TCA in 10 (23%) patients, and with SNRI in 15 (34%) patients.

**FIGURE 3.**
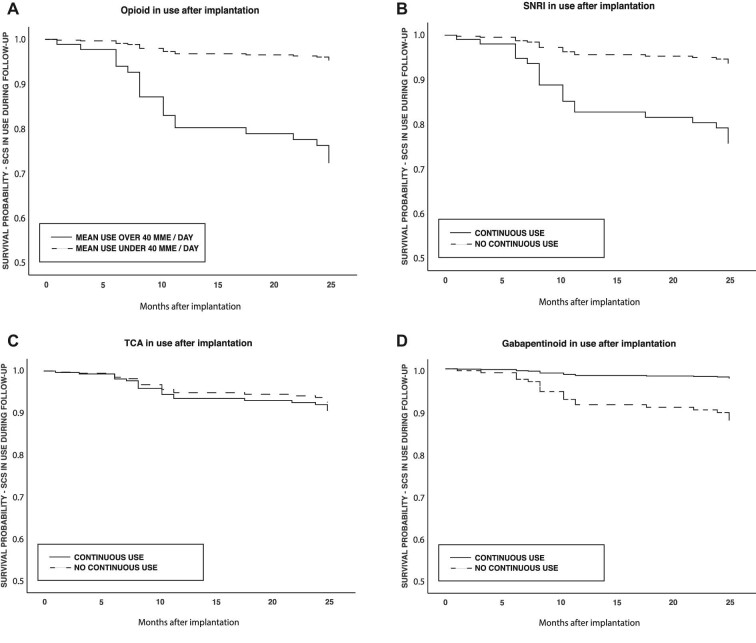
Cox proportional-hazard regression model. Cox proportional-hazard regression model displays survival curve of all 156 patients receiving permanent spinal cord stimulator after trial period. **A**, Opioid over 40 morphine milligram equivalents (MMEs) in use/no opioid or under 40 MMEs in use, **B**, SNRI in use/not in use, **C**, TCA in use/not in use, and **D**, gabapentinoid in use/not in. Medication use was defined as 2 or more purchases during the year after SCS implantation. The end point of follow-up was SCS explantation or the end of the 2-yr follow-up time. Gender, age, number of previous operations, duration and location of pain, status of instrumented lumbar fusion, year of SCS operation (<2004, 2005-2009, >2010), use of benzodiazepines, use of opioid >40 MME/day, use of TCA, use of SNRI, and use of gabapentinoids were used as covariates.

In a multivariate Cox regression, patients using gabapentinoids experienced significantly fewer explantations during the 2-yr follow-up (hazard ratio [HR] 0.18, 95% CI 0.04-0.81, *P = .*026). In contrast, patients with opioid use over 40 MME/day after implantation had significantly more explantations (HR 6.7, 95% CI 2.5-18, *P < .*01) (Table 1 and Figure 3). Explant rate was 12% during 1997 to 2004, when pregabalin was not available, and 10% during 2005 to 2015 with pregabalin available and in use (Tables 1 and 2).

### Effect of Gabapentin Use on Opioid Dose Reduction

Of the 135 patients with SCS in use throughout the follow-up, 89 (66%) were using opioids before implantation, with a mean dose of 33 mg MME/day. Of these 89 patients, 29 (32%) were using gabapentinoids, and 37 (41%) were able to reduce opioid use >50% or discontinue opioids during the follow-up.

Patients using gabapentinoids could significantly more often discontinue opioids or reduce their dose >50% during the 2-yr follow-up (bivariate logistic regression: odds ratio [OR] 5.7, 95% CI 1.4-23, *P = .*015). Patients using opioids over 40 MME/day before SCS implantation were significantly less often able to discontinue opioids or reduce their opioid dose >50% (OR 0.2, 95% CI 0.05-0.87, *P < .*032) (Table 1).

## DISCUSSION

We studied 203 consecutive FBSS patients trialed for SCS in a single tertiary center. Gabapentinoid use was associated with a lower SCS explantation rate during a 2-yr follow-up. Our previous study with the same population showed that opioid use was associated with a higher explantation rate.^19^ Explantations occur most commonly after a patient experiences a loss of SCS efficacy.^12^ It is possible that gabapentinoids protect from this treatment tolerance; this theory needs to be studied with a prospective randomized study.

Gabapentinoid users were able to reduce their opioid dose significantly more often than nonusers. Effects of neuropathic pain medication on SCS outcomes have previously been analyzed in 3 studies (Table 3). Gabapentin and pregabalin did not affect any pain outcomes, but significantly increased the perception of pain (*P* < .001) on the McGill Pain Questionnaire.^20^ Duloxetine improved the affective component of pain 1 yr following surgery, as compared to patients with SCS alone. Neither the use of opioids nor neuropathic pain medications were associated with changes in the odds of a successful SCS trial or a 50% pain reduction.^21^ Quality of life after SCS was better with a combination of opioid and gabapentinoid therapy than with opioid therapy alone.^22^

**TABLE 3.** Summary of Studies of Neuropathic Pain Medication Use in Patients With Spinal Cord Stimulation

Reference Year	Country	Type of study	Cases, groups and follow-up period in years	Mean age ± SD (yr)	Pain disorders included	Medication included	Outcome measure	Outcome
Jang (2018)^22^	Republic of Korea	Retrospective evaluation; medical charts	48 cases Opioid only = 20 Opioid + NP = 28 24 mo postop	Opioid only 60.5 ± 15.5 Opioid + NP 63.5 ± 17.1	CRPS 1, CRPS 2, FBSS, PNP, Raynaud disease, chronic back pain	Gabapentin, pregabalin + morphine, oxycodone, TD fentanyl	NRS, QOLS Data points: 1, 6, 12, 24 mo	A gabapentinoid added to an opioid is superior to an opioid alone in terms of improving QOL
Prabhala (2018)^20^	USA	Prospective follow-up study	108 cases 41 with duloxetine 67 w/o duloxetine 12 mo postop	SCS only 55.6 ± 11.4 SCS + duloxetine 58.0 ± 10.6	FBSS, CRPS, neuropathy	Duloxetine (pregabalin, gabapentin)	NRS, MPQ, BDI, ODI, PCS, GIC Data points: preimplantation, postimplantation 12 mo	Gabapentin and pregabalin did not significantly affect any pain outcomes positively but significantly increased perception of pain (*P <* .001) on MPQ sensory Duloxetine improves the affective component of pain 1 yr following surgery as compared to patients with SCS alone. Further, SCS patients receiving duloxetine were found to be more willing to receive SCS surgery again, and this willingness correlated with increased doses
Maher (2019)^21^	USA	Retrospective cohort study	115 cases with SCS trial 67 gabapentinoid (58.26%) 34 SNRI (29.57%) 27 TCA (23.48%) 91 opioids (79.13%) 12 mo postop	50.52 ± 13.12	FBSS, CRPS, DPN, vascular claudication	Opioids (MEQ), SSRI, SNRI, TCA, gabapentin/pregabalin, baclofen, benzodiazepine	A successful SCS trial, a 50% decrease in opioid use 1 yr after implant	Neither the use of opioids nor neuropathic pain medications were associated with changes in the odds of a successful SCS trial or a 50% pain reduction. A higher dose of chronic opioid use prior to a trial was associated with greater odds of having a 50% reduction in opioid use following implant

BDI, beck depression inventory; DPN, diabetic peripheral neuropathy; GIC, global impression of change; MPQ, mcgill pain questionnaire; NRS, numeric rating scale; ODI, oswestry disability index; PCS, pain catastrophizing scale; PNP, painful diabetic polyneuropathy; QOLS, quality of life scale; SNRI, serotonin and noradrenaline reuptake inhibitors; SSRI, a selective serotonin reuptake inhibitor.

SCS seems to modulate pain from neurotransmitters, through neuroplasticity, to cortical and subcortical neurocircuits. Multiple studies show that SCS attenuates wide dynamic range spinal interneuron hyperexcitability through Aβ-mediated inhibitory control.^23,24^ Many neurotransmitters have been linked to the SCS effect, including inhibitory neurotransmitters (gamma-aminobutyric acid [GABA], acetylcholine, serotonin, and noradrenaline), and excitatory neurotransmitters (glutamate and aspartate).^25-27^ SCS initiates neuropathic pain modulation through a supraspinal-spinal feedback loop and serotonergic descending fibers.^28^ The SCS effect on inhibitory pathways presents a possibility of augmenting pharmacological effects of stimulation.

Our results are in line with previous rodent studies, suggesting that gabapentinoids might have a beneficial effect on SCS that cannot be observed with gabapentinoids alone. Subeffective doses of gabapentinoids combined with SCS significantly attenuated allodynia,^23^ and when studied at a cellular level, a wide dynamic range of neurons showed prominent hyperexcitability. GABA_B_ receptor agonist baclofen, when administered intrathecally, has shown a prominent effect of potentiating the pain suppression effect of SCS in both humans and rodents.^25,29^ A subeffective dose of amitriptyline enhanced the suppressive effect of SCS on mechanical hypersensitivity.^30^ However, among 19 patients using amitriptyline in our study, no significant effects on either opioid dose reduction or SCS explantation rate were observed.

### Limitations of the Study

This was a retrospective study with obvious limitations. Patients who are reliant on gabapentinoid therapy may have different pain pathology (neuropathic) than patients who are more reliant on opioid therapy (nociceptive). In our hospital, SCS is implanted only for neuropathic pain, which is diagnosed by an experienced pain physician and/or neurosurgeon with neuromodulation expertise. Leg pain in FBSS is most likely radicular neuropathic pain, whereas back pain is more often nociceptive. However, in multivariate analyses, pain location was not a significant risk factor for SCS explantation or opioid dose reduction.

We have used hard endpoints, trial success, explantation rates, and opioid dose reduction to determine the SCS outcome. This approach has been used previously in registry-based studies.^31,32^ Moreover, subjective pain questionnaires are not always ideal for analyzing pain with fluctuating characteristics. Psychological, emotional, and functional limitations that change over time may affect the subjective pain perception and reporting.^33^

Medication use was based on nation-wide registry data and is considered more reliable than patient's own report of use, which is more likely to be influenced by the patient-doctor relationship. Pregabalin was not accepted as a licensed medication in Finland before 2004, which affects the total amount of medication used during the study period. Gabapentin was licensed throughout the study. This has been controlled in the multivariate analyses with a time covariate; overall explant rates did not differ before and after 2004. Pregabalin was protected under patent and expensive throughout the study, and we presume that patients with repeated purchases have complied with the medication regiment.

We have studied a well-characterized and homogenous cohort of patients with FBSS. The findings may not be generalizable to other patient groups, including complex regional pain syndrome, where inflammation plays an important role.^34^

In our study, all implantations were made with a surgical paddle lead with tonic stimulation, which was the only waveform used at that time in our practice. Gabapentinoid effect could be different in paresthesia-free stimulation with partially different pain pathways and possibly different pain transmitting cytokines.^35^ This needs to be further studied, preferably in a randomized controlled setting.

## CONCLUSION

The use of gabapentinoids was associated with a lower spinal cord stimulator explantation rate and a higher chance of over 50% opioid reduction. This indicates that patients with SCS may benefit from concomitant use of gabapentinoids. Prospective randomized trials would be warranted to verify this hypothesis.

### Funding

This study is supported by Medtronic, State Research Funding of Kuopio University Hospital, and University of Eastern Finland. All authors are affiliated with the Kuopio University Hospital or University of Eastern Finland.

### Disclosures

Dr Nissen, Ms Ikäheimo, Dr Huttunen, Dr Jyrkkänen, and Dr von und zu Fraunberg have received travel funding from Medtronic. The other author has no personal, financial, or institutional interest in any of the drugs, materials, or devices described in this article. Dr Nissen has also received funding from Finnish Association for the Study of Pain and travel funding from Boston Scientific and Abbott St Jude Medical. Ms Ikäheimo, Dr Huttunen, Dr Jyrkkänen, and Dr von und zu Fraunberg have received travel funding from Abbott St Jude Medical.

## COMMENT

This study suggests that gabapentinoids are associated with reduced SCS explantation rates and lower postprocedure opioid requirements. The authors further suggest that gabapentinoids could somehow confer a protective effect on these patients that enhances their response to SCS therapy.

Another possibility is that these patients represent a subset of patients who are most amenable to multimodal pain therapy and therefore have better outcomes than the entire group as a whole. Perhaps these patients derive partial benefit from several therapies, such as gabapentinoids, antidepressants, opioids, stimulation, etc, that, when cobbled together, yield a successful outcome. Each component, acting alone, is insufficient to provide a successful outcome. Further, each component is not “protective” but instead adds a certain percentage of pain relief, contributing to overall therapy success. When several drug classes are used, the individual drug doses can be kept relatively low compared to the doses used in monotherapy. This may explain why opioid doses were lower in the multimodal therapy patients (ie, those who also received gabapentinoids).

There may have been too few patients receiving antidepressants as part of a multimodal therapy regimen in this study (n = 10) to reveal a significant contribution from this drug class. Perhaps more patients on antidepressants (or baclofen, medical cannabis, etc) would show a similar effect with these medications as with the gabapentinoids in this study.

**Christopher J. Winfree**

*New York, New York, USA*
